# Anti-inflammatory properties of desipramine and fluoxetine

**DOI:** 10.1186/1465-9921-8-35

**Published:** 2007-05-03

**Authors:** Caroline Roumestan, Alain Michel, Florence Bichon, Karine Portet, Maëlle Detoc, Corinne Henriquet, Dany Jaffuel, Marc Mathieu

**Affiliations:** 1 Inserm, U454, Montpellier, F-34295, France; 2 Laboratoire de Pharmacologie et Physiopathologie Expérimentales, Faculté de Pharmacie, Univ Montpellier, Montpellier, F-34090, France; 3 Centre Médical Spécialisé de Pneumologie, 30 boulevard Kennedy, Béziers, F-34500, France; 4 Present address : Laboratoires Macors, Auxerre, F-89000, France; 5 Present address : Inserm, U826, Montpellier, F-34298, France; 6 Present address : Inserm, U844, Montpellier, F-34091, France

## Abstract

**Background:**

Antidepressants are heavily prescribed drugs and have been shown to affect inflammatory signals. We examined whether these have anti-inflammatory properties in animal models of septic shock and allergic asthma. We also analysed whether antidepressants act directly on peripheral cell types that participate in the inflammatory response in these diseases.

**Methods:**

The antidepressants desipramine and fluoxetine were compared in vivo to the glucocorticoid prednisolone, an anti-inflammatory drug of reference. In a murine model of lipopolysaccharides (LPS)-induced septic shock, animals received the drugs either before or after injection of LPS. Circulating levels of tumour necrosis factor (TNF)-α and mortality rate were measured. In ovalbumin-sensitized rats, the effect of drug treatment on lung inflammation was assessed by counting leukocytes in bronchoalveolar lavages. Bronchial hyperreactivity was measured using barometric plethysmography. In vitro production of TNF-α and Regulated upon Activation, Normal T cell Expressed and presumably Secreted (RANTES) from activated monocytes and lung epithelial cells, respectively, was analysed by immunoassays. Reporter gene assays were used to measure the effect of antidepressants on the activity of nuclear factor-κB and activator protein-1 which are involved in the control of TNF-α and RANTES expression.

**Results:**

In the septic shock model, all three drugs given preventively markedly decreased circulating levels of TNF-α and mortality (50% mortality in fluoxetine treated group, 30% in desipramine and prednisolone treated groups versus 90% in controls). In the curative trial, antidepressants had no statistically significant effect, while prednisolone still decreased mortality (60% mortality versus 95% in controls). In ovalbumin-sensitized rats, the three drugs decreased lung inflammation, albeit to different degrees. Prednisolone and fluoxetine reduced the number of macrophages, lymphocytes, neutrophils and eosinophils, while desipramine diminished only the number of macrophages and lymphocytes. However, antidepressants as opposed to prednisolone did not attenuate bronchial hyperreactivity. In vitro, desipramine and fluoxetine dose-dependently inhibited the release of TNF-α from LPS-treated monocytes. In lung epithelial cells, these compounds decreased TNF-α-induced RANTES expression as well as the activity of nuclear factor-κB and activator protein-1.

**Conclusion:**

Desipramine and fluoxetine reduce the inflammatory reaction in two animal models of human diseases. These antidepressants act directly on relevant peripheral cell types to decrease expression of inflammatory mediators probably by affecting their gene transcription. Clinical implications of these observations are discussed.

## Background

It was hypothesized more than 30 years ago that depression involves a deficiency in monoamine neurotransmission. Immune activation may be causally related to these signaling disorders, as inflammatory cytokines have been shown to alter monoamine turnover, decrease activity of presynaptic serotoninergic neurons, and activate serotonin re-uptake from the synaptic cleft [1,2]. According to this hypothesis, therapeutic effects of antidepressants could be at least partly exerted by attenuating brain expression or action of inflammatory cytokines [3,4]. In this line, administration of the tricyclic antidepressant desipramine in rats has been shown to result in a virtual ablation of neuron-derived tumour necrosis factor (TNF)-α [5,6]. Intracerebroventricular microinfusion of TNF-α prevents the efficacy of desipramine while that of TNF-α antibody mimics the therapeutic effect of the antidepressant, providing further evidences that this cytokine plays a key role in the pathogenesis of depression [7].

Interestingly, antidepressants are also able to decrease peripheral inflammation. Chlomipramine, another tricyclic antidepressant, and fluoxetine, a specific inhibitor of serotonin reuptake, reduce oedema induced by the injection of yeast suspension in the rat hind paw [8,9]. Recently, preventive treatment with bupropion-amfebutamone, a noradrenalinedopamine reuptake inhibitor, was shown to reduce TNF-α release and mortality in a murine model of severe sepsis [10]. In these studies, the anti-inflammatory effects of fluoxetine and bupropion involved, at least partly, a central action.

Depression is a common illness with a 17% lifetime prevalence in the general population [11]. Of note, depressive symptoms and disorders seem to be even more common in asthma patients [12,13]. Lifetime rates of depressive disorder of up to 41% have been reported in clinical samples of patients with asthma [14,15]. Thus, the aim of the present study was to determine the effect of desipramine and fluoxetine in two animal models of human inflammatory disorders, namely septic shock and allergic asthma. In the septic shock model, antidepressants were given either preventively or curatively. We report that desipramine and fluoxetine have therapeutic effects and counteract inflammation in these models. Our data further indicate that these antidepressants can directly act on relevant peripheral cell types to decrease expression of inflammatory cytokines. Finally, we show that desipramine and fluoxetine reduce the activity of the transcription factors nuclear factor (NF)-κB and activator protein (AP)-1, which have been shown to control expression of these cytokines [16,17].

## Methods

### Reagents

12-O-tetradecanoyl-phorbol-13-acetate (TPA), lipopolysaccharides (LPS; Escherichia coli serotype 0111:B4), ovalbumin (OVA), aluminium hydroxide, metacholine, prednisolone and desipramine were purchased from Sigma. Fluoxetine was obtained from Tocris. Recombinant human TNF-α was purchased from BD Pharmingen. For in vivo studies, antidepressants and prednisolone were dissolved in saline. For in vitro studies, antidepressants were initially dissolved in absolute ethanol at 10^-2 ^M. Dilutions with medium were freshly made from original stocks to a maximal concentration of 10^-5 ^M as in previous studies ([18] and references therein). No toxic effects of the drugs or solvents were observed at these dilutions as checked by vital staining with trypan blue and lactate dehydrogenase dosage in supernatants.

### Cell culture

A549 human lung epithelial cells were maintained in Ham's F12/Dulbecco's modified Eagle's medium containing 10 % heat-inactivated fœtal calf serum, 100 U/ml penicillin, 100 mg/ml streptomycin and 2 mM glutamine. Human monocytes were obtained by the following procedure. Buffy coats were collected from the blood of healthy donors. Blood mononuclear cells were isolated by density-gradient centrifugation through Ficoll-Hypaque (Pharmacia), suspended in RPMI 1640 medium with 10 % heat-inactivated fœtal calf serum, and seeded in gelatin-coated flasks. After incubation for 30 min at 37°C, serial washes were performed to eliminate non-adherent cells and adherent monocytes were detached with 10 mM EDTA, resuspended in Iscove's modified Eagle's medium containing 10 % heat-inactivated fœtal calf serum. The day before transfection and/or stimulation, cells were seeded in medium containing 5% charcoal/dextran treated foetal calf serum.

### Reporter plasmids

The AP-1-luciferase gene construct -517/+63 Coll Luc consists of the luciferase gene driven by part of the collagenase promoter with its single AP-1 site (gift of Peter Herrlich, Institute of Genetics, Karlsruhe, Germany). The NF-κB-luciferase gene construct 3 × Igκ Cona Luc contains three tandem repeats of the NF-κB response element from the immunoglobulin κ chain linked to the conalbumin minimal promoter and the luciferase gene (gift of Alain Israël, Institut Pasteur, Paris, France). The pJ7-*LacZ *plasmid contains the SV40 early promoter linked to the β-galactosidase gene.

### RANTES and TNF-α immunoassays

Concentrations of Regulated upon Activation, Normal T cell Expressed and presumably Secreted (RANTES) and TNF-α were determined using quantitative sandwich enzyme immuno-assays as described by the manufacturer (R&D Systems).

### RANTES mRNA quantitation

Total cellular RNA was isolated with RNA PLUS (Quantum Biotechnologies). Colorimetric RANTES mRNA quantitation was performed using a Quantikine mRNA kit (R&D Systems).

### Transient transfection and reporter gene assays

100 000 cells per well were serum deprived overnight and transfected with 60 ng of either 3 × Igκ Cona Luc or -517/+63 Coll Luc and 25 ng of pJ7-*LacZ*. These were then stimulated as indicated in the figure legends. Transfection, luciferase and β-galactosidase assays were performed as described previously [19]. Luciferase activity was divided by β-galactosidase activity to normalise values for variations in transfection efficiency.

### LPS-induced inflammation and endotoxic shock in mice

The effect of antidepressants in septic shock was studied on 5-week-old BALB/c mice weighing 17–21 g (Elevage Janvier). To study their protective effect, animals received i.p. prednisolone, desipramine or fluoxetine at 5, 10 and 20 mg/kg or saline 30 min before injection of a lethal dose of LPS (50 mg/kg, i.p.). Ninety min after receiving LPS, blood was quickly collected from the trunk after decapitation. The serum was then isolated by centrifugation after clotting to determine the concentration of TNF-α. Additional groups of mice receiving saline or the test drugs at 20 mg/kg before LPS treatment were observed for survival. To study the curative effect of antidepressants, animals received a lethal infusion of LPS and were treated with prednisolone (20 mg/kg, i.p), desipramine (20 mg/kg, i.p.), fluoxetine (20 mg/kg, i.p.) or saline 4, 8, 12, 24, and 30 h later. Mortality was evaluated daily.

### Measurement of bronchial responsiveness and inflammation in ovalbumin-sensitized rats

Ten-week-old Brown Norway rats were sensitized to ovalbumin as follows: ovalbumin (1 mg/ml) was emulsified with aluminium hydroxide (100 mg/ml) in saline prior to i.p. injection of 1 ml per rat at day 1, 2, 3 and 16. From day 22 to 29, prednisolone, desipramine, fluoxetine or saline were i.p. administered at 10 mg/kg to rats 30 min prior nebulisation with a 1% (w/v) ovalbumin solution during 20 min. Unsensitized controls received i.p. injections of aluminium hydroxide alone. These were then nebulised with saline from day 22 to 28 and challenged with ovalbumin on day 29. Bronchial responsiveness to metacholine (10 mg/ml nebulised during 2 min) was analysed by barometric plethysmography (Emka Technologies) on conscious unrestrained animals 24 h after the last ovalbumin nebulisation. Enhanced pause (Penh), reflecting the resistance to air flow, and hence airway obstruction, was measured. Rats were then anaesthetized with pentobarbital and exsanguinated by catheterization of the abdominal aorta to avoid contamination of bronchoalveolar lavages with red cells. The trachea was stripped and rinsed in situ with PBS. Total number of cells in bronchoalveolar lavages was immediately determined by counting on Malassez chamber. The different cell types were distinguished and counted after cytocentrifugation, fixation and May Grünwald Giemsa staining. These experimentations have been carried out in accordance with the Declaration of Helsinki and with the Guide for the Care and Use of Laboratory Animals as adopted and promulgated by the US National Institutes of Health. Our laboratory practice was approved by the « Comité Régional d'Ethique pour l'Expérimentation Animale du Languedoc-Roussillon ».

### Statistical analysis

Data obtained in vitro are presented as mean ± SE of at least three independent experiments performed in duplicates. Analysis of in vivo experiments was done on 6 to 31 animals per treatment group. Data were analysed using the Instat software (GraphPad Software, San Francisco, CA). Statistical significance was set up at p < 0.05.

## Results

### Pretreatment with desipramine and fluoxetine prevent LPS-induced systemic inflammation and mortality in mice

**Figure 1:**
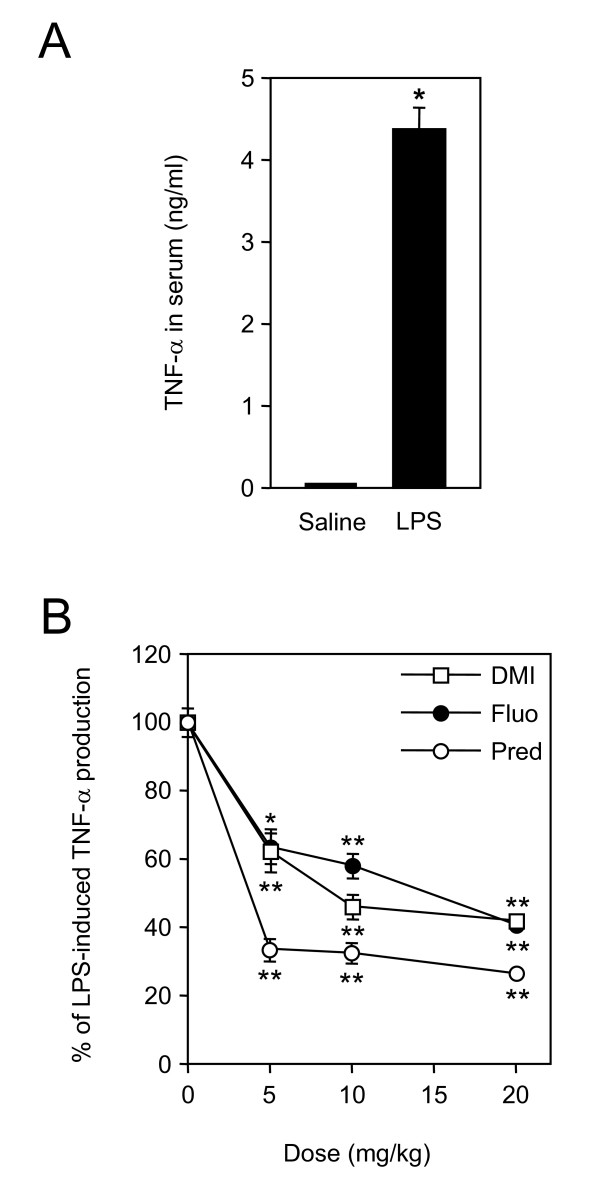
**Desipramine and fluoxetine inhibit release of TNF-α in serum of LPS-treated mice**. A, Mice (n = 12 per group) were injected with saline or LPS (50 mg/kg, i.p.). After 90 min, blood was collected and serum concentration of TNF-α was measured. Concentration was 38 ± 12 pg/ml in saline and 4364 ± 265 pg/ml in LPS-treated mice. *p < 0.0001 versus saline, by Welch t test. B, Mice (n = 6–12 per group) were i.p. injected with saline or the indicated doses of desipramine, fluoxetine or prednisolone 30 min before administration of LPS. Blood was collected 90 min after LPS treatment, and serum concentration of TNF-α was measured. Data are shown as the percentage of LPS-induced TNF-α release. *p < 0.05 and **p < 0.001 versus LPS alone, by ANOVA with Bonferroni post-test.

**Figure 2:**
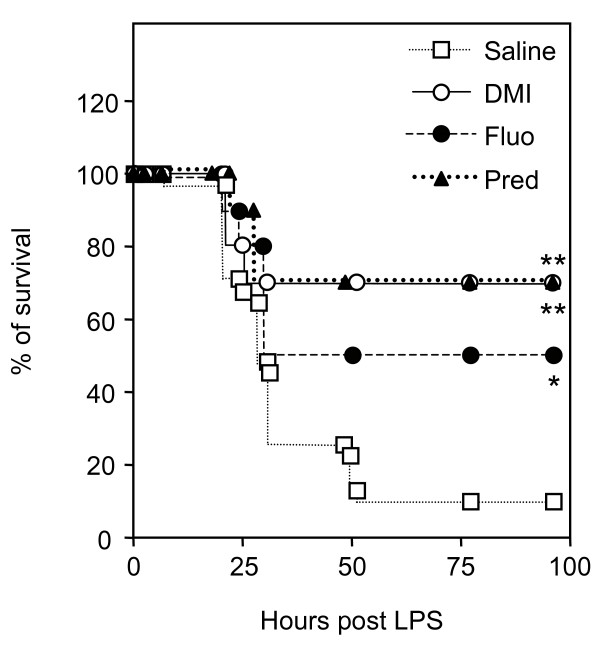
**Pretreatment with desipramine and fluoxetine prevents LPS-induced mortality**. Mice (n = 10–31 per group) were injected with saline, desipramine (DMI, 20 mg/kg, i.p.), fluoxetine (Fluo, 20 mg/kg, i.p.) or prednisolone (Pred, 20 mg/kg, i.p.) as indicated 30 min before administration of LPS (50 mg/kg, i.p.). Kaplan-Meier survival curves represent the percentage of surviving individuals in each groups. Survival is shown over a 96 h period (no additional death occurred after 96 h). *p = 0.0129 and **p = 0.0005 versus saline, by Fisher's exact test.

The protective effect of desipramine and fluoxetine was tested in a murine model of septic shock, in which endotoxemia and systemic inflammation is triggered by LPS injection. Production of TNF-α is one of the earliest events induced by LPS. Indeed, upon LPS treatment, concentration of TNF-α in the serum reached a peak after 90 min and was back to basal level after 3 h (Fig. 1A and data not shown). Therefore, mice were pretreated with the test compound or saline 30 min before injection of LPS and TNF-α concentration was measured 90 min afterwards. Significant inhibition of TNF-α production occurred with either drug at 5 mg/kg. This dose corresponds to the recommended daily dosage of desipramine in humans. Because the inhibitory effect of prednisolone, desipramine and fluoxetine was stronger at 10 or 20 mg/kg, these doses were used in following experiments (Fig. 1B). We next analysed the survival of mice injected with LPS and pretreated with 20 mg/kg of prednisolone, desipramine or fluoxetine. These compounds notably increased survival. The proportion of living mice rose from 10% in the control group to 50% in the fluoxetine group, and to 70% in the prednisolone and desipramine groups (Fig. 2).

### Effect of curative treatment with desipramine and fluoxetine on LPS-induced mortality in mice

**Figure 3:**
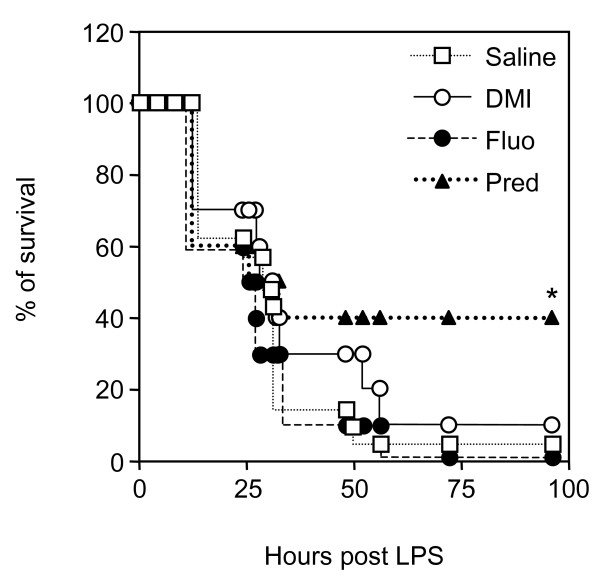
**Effect of a curative treatment with desipramine or fluoxetine on LPS-induced mortality**. Mice (n = 10–21 per group) were treated with prednisolone (Pred, 20 mg/kg, i.p), desipramine (DMI, 20 mg/kg, i.p.), fluoxetine (Fluo, 20 mg/kg, i.p.) or saline 4, 8, 12, 24, and 30 h after administration of LPS (50 mg/kg, i.p.) as indicated. Kaplan-Meier survival curves represent the percentage of surviving individuals in each groups. Mortality was evaluated daily (no additional death occurred after 96 h). *p = 0.0274 versus saline, by Fisher's exact test.

Because severe sepsis is an acute inflammatory syndrome, specific treatment in the clinic is initiated after symptoms have been declared. To mimic such curative treatment in mice, repeated injections of antidepressants or prednisolone were performed, starting 4 h after administration of LPS, a time at which the first signs of sepsis such as diarrhoea, hypoactivity, piloerection, and shivering are apparent. Prednisolone significantly increased the survival of mice (40% survival versus 5% in the control group) (Fig. 3). Fluoxetine had no curative effect while desipramine delayed time of death by few hours (Fig. 3). However, this latter effect was not statistically significant.

### Desipramine and fluoxetine inhibit release of TNF-α from freshly isolated human monocytes

**Figure 4:**
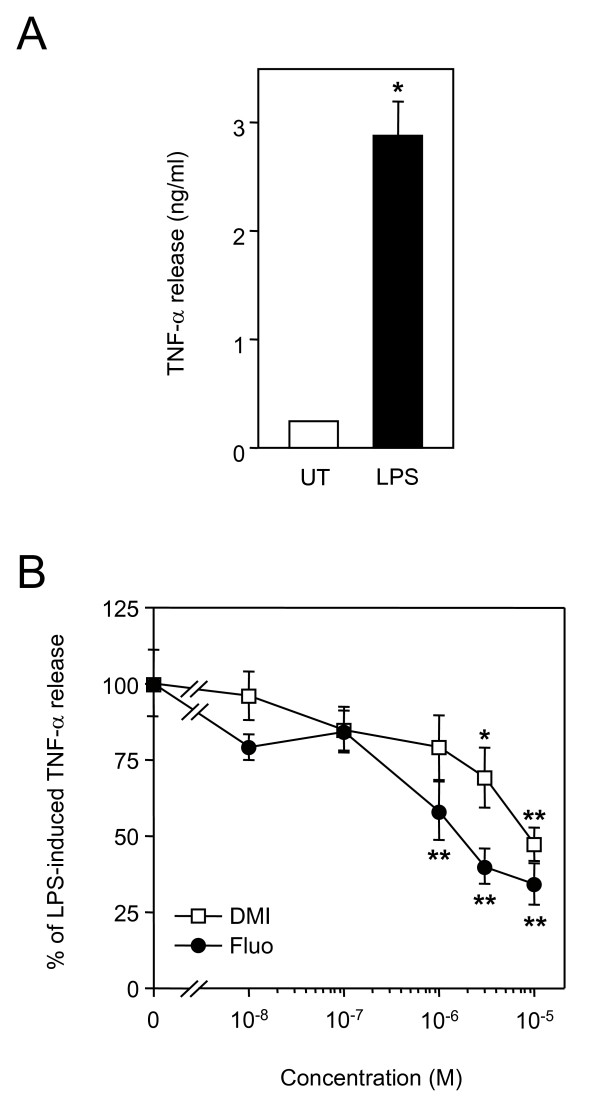
**Desipramine and fluoxetine inhibit release of TNF-α from cultured monocytes**. A, Purified human monocytes were seeded at 100 000 cells per well and were left untreated (UT) or stimulated for 20 h with 100 ng/ml of LPS. Concentration of TNF-α in supernatants was then measured. *p < 0.0001 versus untreated, by Welch t test. B, Cells were treated for 20 h with 100 ng/ml of LPS in the presence of increasing concentrations of desipramine (DMI) or fluoxetine (Fluo). Data are shown as the percentage of LPS-induced TNF-α release. *p < 0.05 and **p < 0.001 versus LPS alone, by ANOVA with Bonferroni post-test.

Monocytes are the main source of TNF-α produced in the blood stream during septic shock. Therefore, we next tested whether desipramine and fluoxetine could act directly on monocytes to inhibit LPS-induced TNF-α release. After LPS treatment of human monocytes in primary cultures, concentration of TNF-α in supernatants raised from 226 ± 16 pg/ml to 2848 ± 309 pg/ml (Fig. 4A). As shown in Fig. 4B, desipramine and fluoxetine dose-dependently inhibited TNF-α release.

### Desipramine and fluoxetine reduce bronchial inflammation but not hyper responsiveness in ovalbumin-sensitized rats

**Figure 5:**
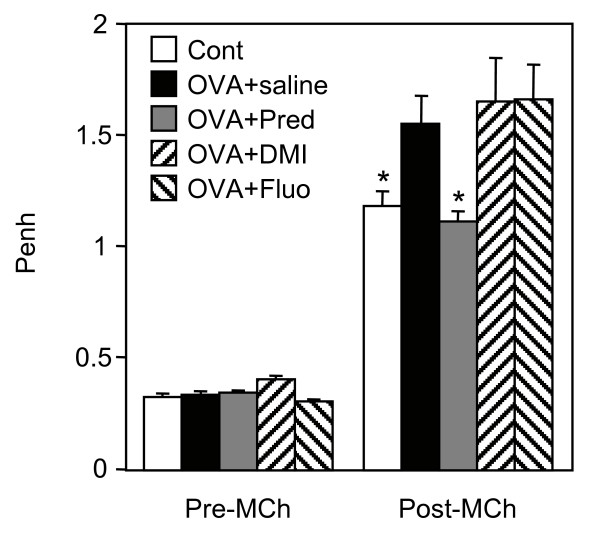
**Desipramine and fluoxetine do not reduce bronchial hyper responsiveness in sensitized rats**. Unsensitized control rats (Cont) were nebulised with ovalbumin. Ovalbumin-sensitized rats were treated with saline (OVA+saline), prednisolone (OVA+Pred), desipramine (OVA+DMI) or fluoxetine (OVA+Fluo) at 10 mg/kg prior to nebulisation with ovalbumin. Rats (n = 6–7 per group) were then challenged with metacholine 24 h after ovalbumin nebulisation. Enhanced pause (Penh), which reflects airway obstruction, was measured by barometric plethysmography before (Pre-Mch) and after (Post-Mch) the challenge. * p < 0.05 versus OVA+saline, by Student t test.

**Figure 6:**
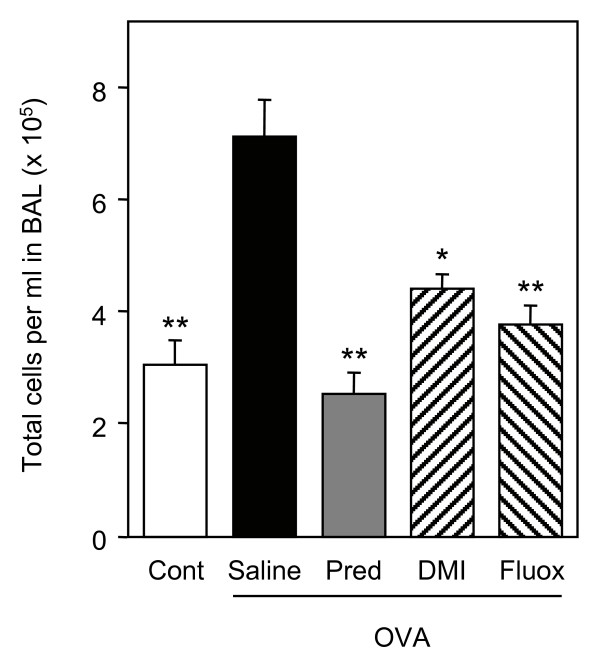
**Desipramine and fluoxetine inhibit influx of inflammatory cells in bronchoalveolar lavages of sensitized rats**. Ovalbumin-sensitized rats (OVA) treated with saline, prednisolone (Pred, 10 mg/kg), desipramine (DMI, 10 mg/kg) or fluoxetine (Fluo, 10 mg/kg) and control rats (Cont) (n = 6–7 per group) were challenged with metacholine. Total number of inflammatory cells in bronchoalveolar lavages was then determined. *p < 0.01 and **p < 0.001 versus ovalbumin-sensitized rats treated with saline, by Student t test.

**Figure 7:**
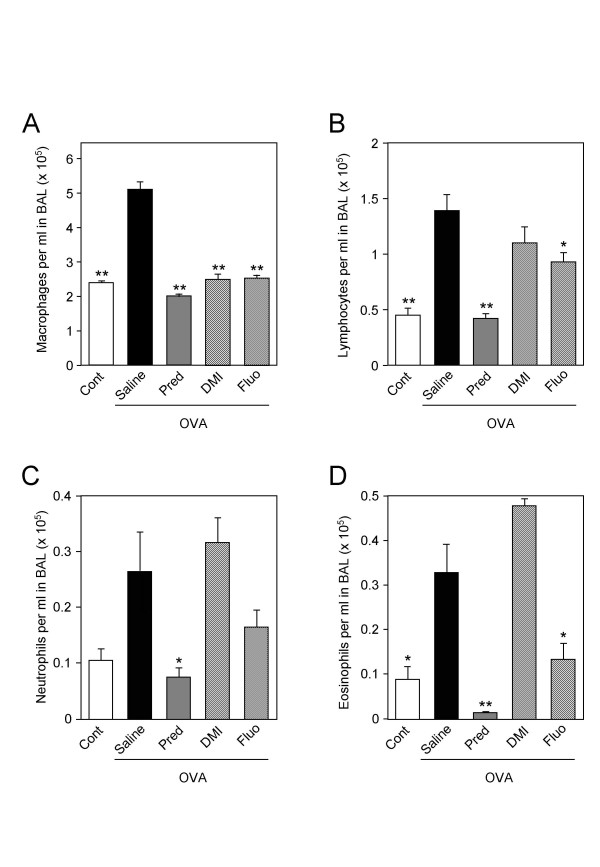
**Effects of desipramine and fluoxetine on leukocyte sub-populations in bronchoalveolar lavages of sensitized rats**. Ovalbumin-sensitized rats (OVA) treated with saline, prednisolone (Pred, 10 mg/kg), desipramine (DMI, 10 mg/kg) or fluoxetine (Fluo, 10 mg/kg) and control rats (Cont) (n = 6–7 per group) were challenged with metacholine. Cells in bronchoalveolar lavages were spun down on cytoslides, fixated and stained. Macrophages (panel A), lymphocytes (panel B), neutrophils (panel C) and eosinophils (panel D) were then counted under a microscope. A minimum of 200 cells was counted for each bronchoalveolar lavage. *p < 0.05 and **p < 0.001 versus ovalbumin-sensitized rats treated with saline, by Student t test.

The anti-inflammatory effects of antidepressants were also analysed in an animal model of allergic asthma. Brown Norway rats were sensitized to ovalbumin to trigger bronchial hyperresponsiveness and airway inflammation. The increase in bronchial responsiveness to metacholine observed in sensitized rats was significantly reduced by prednisolone but not by desipramine or fluoxetine (Fig. 5). Bronchoalveolar lavages of sensitized rats contained an increased number of total inflammatory cells, which was markedly inhibited by the three drugs (Fig. 6). Analysis of leukocytes sub-populations revealed that prednisolone and fluoxetine reduced the number of macrophages (by 60% and 51%, respectively), lymphocytes (by 70% and 33%, respectively), neutrophils (by 72% and 38%, respectively) and eosinophils (by 97% and 60%, respectively), while desipramine diminished only the number of macrophages (by 52%) and lymphocytes (by 21%) (Fig. 7).

### Desipramine and fluoxetine inhibit expression of RANTES in lung A549 epithelial cells

**Figure 8:**
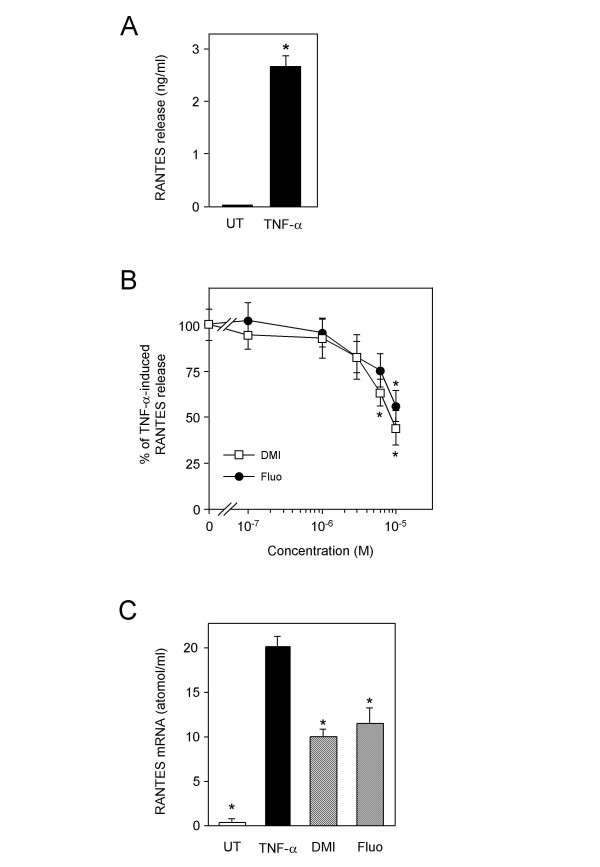
**Desipramine and fluoxetine inhibit RANTES expression**. A, A549 cells were left untreated (UT) or stimulated for 20 h with TNF-α at 10 ng/ml. Concentration of RANTES in supernatants was then measured. *p < 0.0001 versus untreated, by Welch t test. B, A549 cells were stimulated for 20 h with 10 ng/ml of TNF-α in the presence of increasing concentrations of desipramine or fluoxetine. Data are shown as the percentage of TNF-α-induced RANTES release. *p < 0.05 versus TNF-α alone, by ANOVA with Bonferroni post-test. C, A549 cells were either left untreated (UT) or pretreated or not for 1 h with desipramine (DMI) or fluoxetine (Fluo) at 10^-5 ^M and further stimulated for 4 h with TNF-α at 10 ng/ml. The amount of RANTES mRNA was quantified by a colorimetric assay. *p < 0.05 versus TNF-α alone, by Student t test.

Accumulation of inflammatory cells in bronchoalveolar lavages may result, at least in part, from an effect on lung epithelial cells which have the capacity to produce chemokines. Thus, we examined the effect of desipramine and fluoxetine on RANTES production in A549 lung epithelial cells. After TNF-α treatment, concentration of RANTES in cell culture supernatants raised from 7 ± 5 pg/ml to 2659 ± 227 pg/ml (Fig. 8A). Antidepressants dose dependently inhibited TNF-α-induced RANTES release. A maximal inhibition of 50% was obtained at 10^-5 ^M (Fig. 8B). TNF-α-induced RANTES transcripts accumulation was also decreased by approximately 50% after treatment by either antidepressant (Fig. 8C).

### Desipramine and fluoxetine repress NF-κB and AP-1 activities

**Figure 9:**
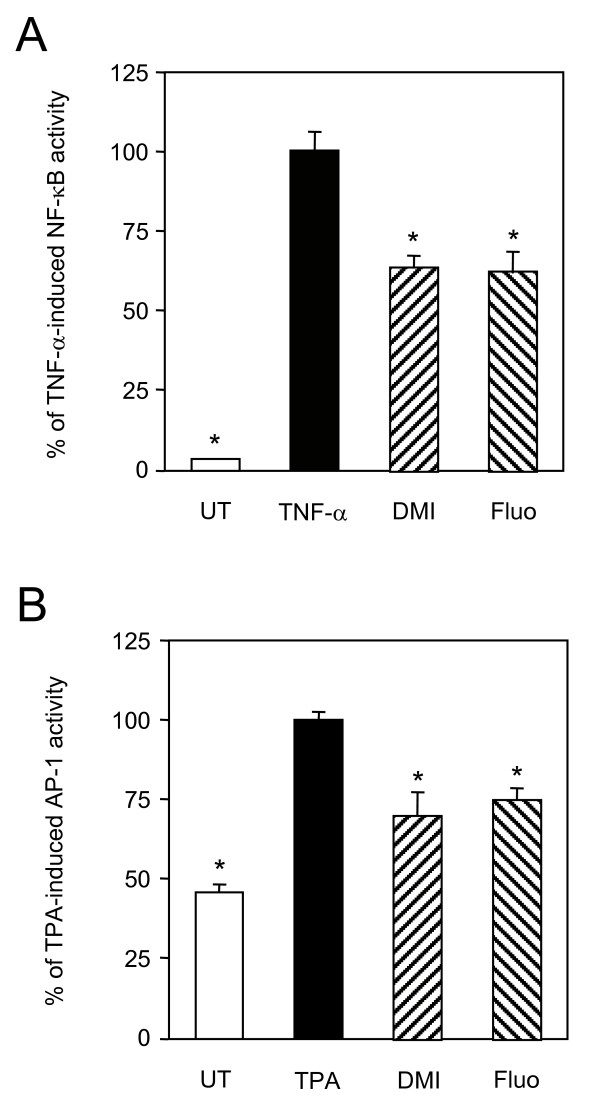
**Desipramine and fluoxetine repress NF-κB and AP-1 activities**. A, A549 cells were transfected with a NF-κB luciferase gene construct and pJ7-*LacZ*. After transfection, cells were either left untreated (UT) or pretreated or not for 1 h with desipramine (DMI, 10^-5 ^M) or fluoxetine (Fluo, 10^-5 ^M), and further stimulated for 4 h with TNF-α (10 ng/ml). NF-κB activity was normalised and that induced by TNF-α alone (47 ± 8 fold induction) was given the nominal value of 100 %. Data are shown as the percentage of activity relative to this nominal value. *p < 0.001 versus TNF-α alone, by Welch t test. B, A549 cells were transfected with an AP-1 luciferase gene construct and pJ7-*LacZ*. After transfection, cells were either left untreated (UT) or pretreated or not for 1 h as in A, and further stimulated for 4 h with TPA (10 ng/ml). AP-1 activity was normalised and that induced by TPA alone (2.3 ± 0.1 fold induction) was given the nominal value of 100 %. Data are shown as the percentage of activity relative to this nominal value. *p < 0.01 versus TPA alone, by Welch t test.

Because NF-κB and AP-1 play a crucial role in the expression and action of inflammatory mediators such as TNF-α and RANTES, activity of these transcription factors was measured in A549 cells treated by desipramine or fluoxetine. Both antidepressants significantly repressed TNF-α-induced NF-κB activity by about 40% (Fig. 9A). Desipramine and fluoxetine decreased TPA-induced AP-1 activity by 30% and 25%, respectively (Fig. 9B).

## Discussion

### Central versus peripheral action

In the present study, desipramine and fluoxetine are shown to affect the capacity of monocytes and lung epithelial cells to produce inflammatory cytokines in vitro. This observation favours a direct peripheral anti-inflammatory action of antidepressants. However, fluoxetine but not the desipramine-related compound chlomipramine has been shown to trigger anti-inflammatory effects through the potentiation of serotoninergic transmission ending up in activation of the pituitary-adrenocortical axis [8,9]. Further evidence for a central mechanism was provided for bupropion-amfebutamone in a murine model of severe sepsis. In this study, β-adrenergic and dopaminergic receptor antagonists partially prevented bupropion from reducing mortality rate [10]. To determine the relative contribution of central and peripheral action in the anti-inflammatory effects, it would be interesting to modify antidepressant molecules so that they do not cross the blood-brain barrier. If active, such molecules could become prototypes for new anti-inflammatory drugs.

### Molecular targets of antidepressants

The few previous reports investigating the effects of antidepressants on AP-1 and NF-κB were performed with brain tissues or a neuronal cell line. Antidepressants were found to regulate either positively or negatively DNA binding activity of these transcription factors, depending on the drug's chemical class, the brain region, and on whether administration was acute or chronic [20-22]. We report that desipramine and fluoxetine repress NF-κB and AP-1 activities in a lung epithelial cell line. This may account for their anti-inflammatory effects in the animal model of asthma as observed here. Indeed, AP-1 and NF-κB have been involved in the pathogenesis of various chronic inflammatory diseases, including asthma and allergy [23,24]. In addition, NF-κB plays a key role in the mortality of sepsis [25]. Thus, repression of NF-κB activity by antidepressants may also explain their protective effect in the model of septic shock. Concentrations of 1 to 10 μM were required to down-regulate AP-1 and NF-κB activities and expression of inflammatory cytokines. Such concentrations of antidepressants are reached in the plasma [26,27], but are 10 to 1000 times higher than the dissociation constant of antidepressants for monoamine transporters and receptors. Moreover, monoamine transporters and receptors recognized by desipramine and fluoxetine are not expressed in A549 cells as checked using oligonucleotide microarrays (data not shown). Hence, antidepressants should exert their anti-inflammatory effects through lower affinity receptors or effectors that remain to be identified. Interestingly, fluoxetine at 5 to 15 μM was shown to interfere with the activity of extrusion pumps [28]. Similarly, desipramine at 10 μM was found to reduce P-glycoprotein-like activity in vitro, thereby enhancing glucocorticoid action [18]. As opposed to this latter study, in our in vitro assays, antidepressants produced antiinflammatory effects in the complete absence of glucocorticoid. Moreover, A549 cells do not express P-glycoprotein [29]. The effects of antidepressants in these cells are thus not mediated through modulation of P-glycoprotein. Nevertheless, inhibition of drug efflux transporters in vivo should increase concentration of endogenous glucocorticoids some in target cells and may account, at least partly, for the anti-inflammatory properties of antidepressants.

### Therapeutic effect of desipramine and fluoxetine in models of inflammatory diseases

The data presented herein demonstrate that desipramine and fluoxetine have significant antiinflammatory properties in animal models of allergic asthma and septic shock. Preventive treatment with desipramine and fluoxetine markedly reduced TNF-α production and mortality in the LPS-induced septic shock model. Desipramine provided the same protection against mortality as the glucocorticoid prednisolone. However, it was less potent than prednisolone in reducing TNF-α production. It is not surprising that the inhibitory effect on TNF-α production is not strictly correlated with survival rate since TNF-α is not the sole mediator of lethality in severe sepsis [30]. Recently, another antidepressant, bupropion-amfebutamone, was also shown to have a preventive therapeutic effect in a murine model of severe sepsis [10]. In the same model, we further show that antidepressants administered curatively do not reduce final mortality rate, although desipramine seem to delay time of death by few hours.

In the model of allergic asthma, antidepressants reduced lung inflammation but not bronchial hyper responsiveness, whereas prednisolone was active on both aspects of the pathology. Moreover, both antidepressants were not as efficient as prednisolone in inhibiting inflammatory cell counts. Thus, desipramine and fluoxetine exert weaker or more restricted anti-inflammatory effects compared to those of a glucocorticoid. Moreover, differences were noted between the anti-inflammatory potencies of desipramine and fluoxetine. Desipramine did not reduce lung infiltration of neutrophils and eosinophils as opposed to fluoxetine. Yet, in cultured lung epithelial cells, both antidepressants inhibited with similar efficacy the production of RANTES, a known chemotactic for eosinophils. Possibly, expression of other eosinophils chemoattractants is affected by fluoxetine but not by desipramine in the ovalbumin-sensitized rat model. Our observation made in this model is also in apparent contradiction with data obtained by others showing an inhibition of neutrophils migration by tricyclic antidepressants but not by fluoxetine [31]. However, in this latter study, the migration experiments were performed with neutrophils in vitro. In vivo, antidepressants have a different effect probably because they act on various additional cell types such as T cells, macrophages, endothelial and epithelial cells that affect the migration process.

## Conclusion

The observation that antidepressants have anti-inflammatory properties might have important clinical implications since these drugs are heavily prescribed worldwide and chronic treatment often lasts several months. In France alone, over 11 millions prescriptions for antidepressants were made during year 2000 [32]. Moreover, there is a high prevalence of depression in patients with asthma or other chronic inflammatory diseases [12,13,33]. Thus, the anti-inflammatory effects of antidepressants should be considered especially in depressive patients with inflammatory co-morbidity. In this regard, tianeptine and citalopram, two antidepressants with yet opposite action on serotonin re-uptake, were shown to provide clinical benefit in asthma. In asthmatic children, tianeptine reduces asthma symptoms and increases pulmonary function [34], while in patients with asthma and major depressive disorder, citalopram decreases systemic glucocorticoid use, an important measure of severe asthma exacerbations [35]. Tianeptine decreases free serotonin plasma levels which are high in symptomatic patients with asthma, and seems thus to act as a bronchodilator. Obviously, another mechanism underlies the anti-inflammatory properties of desipramine and fluoxetine since these drugs do not reduce bronchial hyperresponsiveness as shown herein. To ascertain the significance of their anti-inflammatory effects in humans, further clinical trials with antidepressants are required as well as retrospective epidemiological studies assessing the prevalence of inflammatory disorders, including septic shock, in antidepressant-treated subjects.

## Abbreviations

IκB, inhibitor of nuclear factor-κB; IKK, inhibitor of nuclear factor-κB kinase; i.p., intraperitoneally; LPS, lipopolysaccharides; NF-κB, nuclear factor-κB; OVA, ovalbumin; PBMC, peripheral blood mononuclear cells; Penh, enhanced pause; RANTES, Regulated upon Activation, Normal T cell Expressed and presumably Secreted; TNF-α, tumour necrosis factor-α; TPA, 12-O-tetradecanoyl-phorbol-13-acetate.

## Competing interests

The author(s) declare that they have no competing interests.

## Authors' contributions

CR conceived of the study, acquired and analysed most of the data. AM participated in the conception of the in vivo experiments. AM, FB, KP and MD helped to carry out the in vivo assays. CH helped to carry out the in vitro assays. DJ participated in the study design and revised the manuscript critically. MM coordinated the study, participated in the acquisition, analysis and interpretation of the data and drafted the manuscript. All authors read and approved the final manuscript.
